# Copresence Was Found to Be Related to Some Pupil Measures in Persons With Hearing Loss While They Performed a Speech-in-Noise Task

**DOI:** 10.1097/AUD.0000000000001361

**Published:** 2023-04-04

**Authors:** Hidde Pielage, Bethany J. Plain, Gabrielle H. Saunders, Niek J. Versfeld, Thomas Lunner, Sophia E. Kramer, Adriana A. Zekveld

**Affiliations:** 1 Amsterdam UMC location Vrije Universiteit Amsterdam, Otolaryngology-Head and Neck Surgery, section Ear & Hearing, Amsterdam, the Netherlands; 2 Eriksholm Research Centre, Snekkersten, Denmark; 3 Manchester Centre for Audiology and Deafness, University of Manchester, Manchester, United Kingdom.

**Keywords:** Copresence, Effort, Hearing loss, Pupil dilation response, Speech-in-noise, Speech perception

## Abstract

**Objectives:**

To assess if a manipulation of copresence was related to speech-in-noise task performance, arousal, and effort of persons with hearing loss. Task-related arousal and effort were measured by means of pupillometry.

**Design:**

Twenty-nine participants (mean age: 64.6 years) with hearing loss (4-frequency pure-tone average [4F-PTA] of 50.2 dB HL [SD = 8.9 dB] in the right ear and 51.3 dB HL [SD = 8.7 dB] in the left ear; averaged across 0.5, 1, 2, and 4 kHz) listened to and repeated spoken Danish sentences that were masked by four streams of continuous speech. Participants were presented with blocks of 20 sentences, during which copresence was manipulated by having participants do the task either alone or accompanied by two observers who were recruited from a similar age group. The task was presented at two difficulty levels, which was accomplished by fixing the signal-to-noise ratio of the speech and masker to match the thresholds at which participants were estimated to correctly repeat 50% (difficult) or 80% (easy) of the sentences in a block. Performance was assessed based on whether or not sentences were repeated correctly. Measures of pupil size (baseline pupil size [BPS], peak pupil dilation [PPD], and mean pupil dilation [MPD]) were used to index arousal and effort. Participants also completed ratings of subjective effort and stress after each block of sentences and a self-efficacy for listening-questionnaire.

**Results:**

Task performance was not associated with copresence, but was found to be related to 4F-PTA. An increase in BPS was found for copresence conditions, compared to alone conditions. Furthermore, a post-hoc exploratory analysis revealed that the copresence conditions were associated with a significantly larger pupil size in the second half of the task-evoked pupil response (TEPR). No change in PPD or MPD did was detected between copresence and alone conditions. Self-efficacy, 4F-PTA, and age were not found to be related to the pupil data. Subjective ratings were sensitive to task difficulty but not copresence.

**Conclusion:**

Copresence was not found to be related to speech-in-noise performance, PPD, or MPD in persons with HL but was associated with an increase in arousal (as indicated by a larger BPS). This could be related to premobilization of effort and/or discomfort in response to the observers’ presence. Furthermore, an exploratory analysis of the pupil data showed that copresence was associated with greater pupil dilations in the second half of the TEPR. This may indicate that participants invested more effort during the speech-in-noise task while in the presence of the observers, but that this increase in effort may not necessarily have been related to listening itself. Instead, other speech-in-noise task-related processes, such as preparing to respond, could have been influenced by copresence.

## INTRODUCTON

Typically, real-life listening takes place in the presence of others (copresence). This raises the question of whether copresence influences how people listen, in particular with regards to the effort invested and resulting performance. It has been well documented that copresence can influence performance on a plethora of tasks (e.g., Zajonc 1965; Sanders et al. 1978; Buck et al. 1992; Guerin 1993). This phenomenon has been related to changes in (the motivation to exert) effort (McFall et al. 2009; Belletier et al. 2019) and has been commonly referred to as “social facilitation and inhibition” (Belletier et al. 2019). A meta-analysis by Bond and Titus (1983) summarizes the effects of copresence. Based on 241 studies the authors drew several conclusions: (1) during easy tasks, speed (but not performance) improved while others were copresent. (2) During difficult tasks, copresence resulted in both speed and performance decrements. (3) Heart rate and skin conductance measures differed between copresence and alone conditions, but only when tasks were difficult. While a myriad of tasks (e.g., word association, motor, and memory) have been used to study the effects of copresence (many of which were summarized in the meta-analysis by Bond and Titus), it is not known if copresence is associated with performance or effort during listening.

To assess if copresence was related to listening performance and effort, Pielage et al. (2021) had normal hearing participants perform a standard speech-in-noise (SiN) test either alone or together with another participant in a turn-taking fashion. While not fully reflecting a scenario encountered in daily-life, this design allowed for the investigation of the relationship between copresence and listening in a controlled manner. The experiment showed that while participants were in the presence of another participant, listening evoked greater peak pupil dilations (PPDs) compared to when participants completed the task alone. This was interpreted to indicate that participants invested more effort when another person was copresent and participating in the task. However, the increase of effort was not related to a change in SiN performance.

The study by Pielage et al. (2021) included only participants with normal hearing. It is plausible that the relationship between copresence and listening might be stronger for persons with hearing loss (HL) because HL makes speech understanding more difficult and potentially leads to low hearing-related self-efficacy, defined as the belief that one can manage a listening situation given one’s hearing capabilities (Jennings et al. 2014). As copresence interacts with the (experienced) difficulty of the task (Bond & Titus 1983), the relationship between copresence and listening could be stronger in persons with HL. Furthermore, HL and copresence independently impose demands on working memory, attention and effort, which might strengthen the relationship between copresence and listening even further (Shinn-Cunningham 2007; Wagstaff et al. 2008; Zekveld et al. 2011; Rudner et al. 2011; Pichora-Fuller 2016; Belletier et al. 2019).

The current study explored if SiN task-related effort and performance were related to copresence in participants with HL. To do so, the study had participants perform a SiN task at different levels of difficulty, both alone and in the presence of two observers. This design differed from that used by Pielage et al. (2021), where participant took turns while performing the same task. As a result of the turn-taking in the previous experiment, the observed associations could not be exclusively attributed to copresence, as competitiveness might also have played a role. Therefore, to eliminate this, the current study manipulated copresence by means of two observers who did not participate in the task.

Effort related to listening can be measured behaviorally with dual-task paradigms (Gagné et al. 2017), by using physiological measures (Pichora-Fuller 2016; Zekveld et al. 2018; Plain et al. 2020) and via self-report (Picou & Ricketts 2014). In this study, we measured effort related to the listening task by means of pupil measurements. Specifically, we measured PPD and mean pupil dilation (MPD) (for a review, see Zekveld et al. 2018). These pupil measures were further complemented by considering baseline pupil size (BPS), which informed about arousal before the onset of target stimuli (Wang et al. 2018; Ayasse & Wingfield 2020). Here, arousal is defined as it was in the framework for effortful listening (Pichora-Fuller et al. 2016, p. 11S), namely: “a fundamental property of behavior, related to phenomena such as sleep, attention, anxiety, stress, and motivation,” which is based on the work of Aston-Jones and Cohen (2005). This anticipatory state of arousal has been related to preemptive allocation of effort to prepare for the task at hand (Koelewijn et al. 2014; Pichora-Fuller et al. 2016) and task-related anxiety (e.g., reduced confidence that one will successfully complete the task) (Lempert et al. 2015). In addition to pupillometry, measures of self-reported effort and stress were included to determine whether these were related to task difficultly and/or copresence as well. Furthermore, it was assessed if listening-related self-efficacy interacted with copresence effects.

We hypothesized that (1) there would be a significant interaction between copresence and task difficulty on SiN performance, such that performance would be poorer in copresence conditions when the task was difficult, but not when it was easy. (2) The copresence manipulation would be associated with an increase in PPD and MPD (McFall et al. 2009; Belletier et al. 2019; Pielage et al. 2021). (3) The copresence manipulation would not be associated with a change in BPS. (4) The task difficulty manipulation, but not the copresence manipulation, would be associated with a change in subjective ratings of effort. (5) The relationship between the copresence manipulation and pupil measures would be negatively associated with self-efficacy.

## METHODS

### Participants

The participant database at Eriksholm Research Centre (Denmark) was used to recruit 29 participants with HL (17 males, 12 females; aged 47 to 76 years [mean 64.6, SD 9.1]) between October 22, 2019 and December 10, 2019. Participants were required to be native Danish speakers, current hearing aid users and meet the following criteria: (1) a minimum 4-frequency pure-tone average (4F-PTA) of 35 dB HL in both ears, with 4F-PTA defined as the average of thresholds at 0.5, 1, 2, and 4 kHz; (2) symmetrical HL (<15 dB difference between left and right ear at 0.5, 1, and 2 kHz, and <30 dB at 3, 4, and 6 kHz); (3) no history of neurological or psychiatric diseases, use of psychoactive drugs, eye diseases, or diabetes; and (4) no pacemaker. The latter criteria was included as participants were also attached to a device measuring cardiovascular activity (data reported elsewhere: Plain et al. 2021), which could have posed a risk to pacemaker users.

**Fig. 1.**
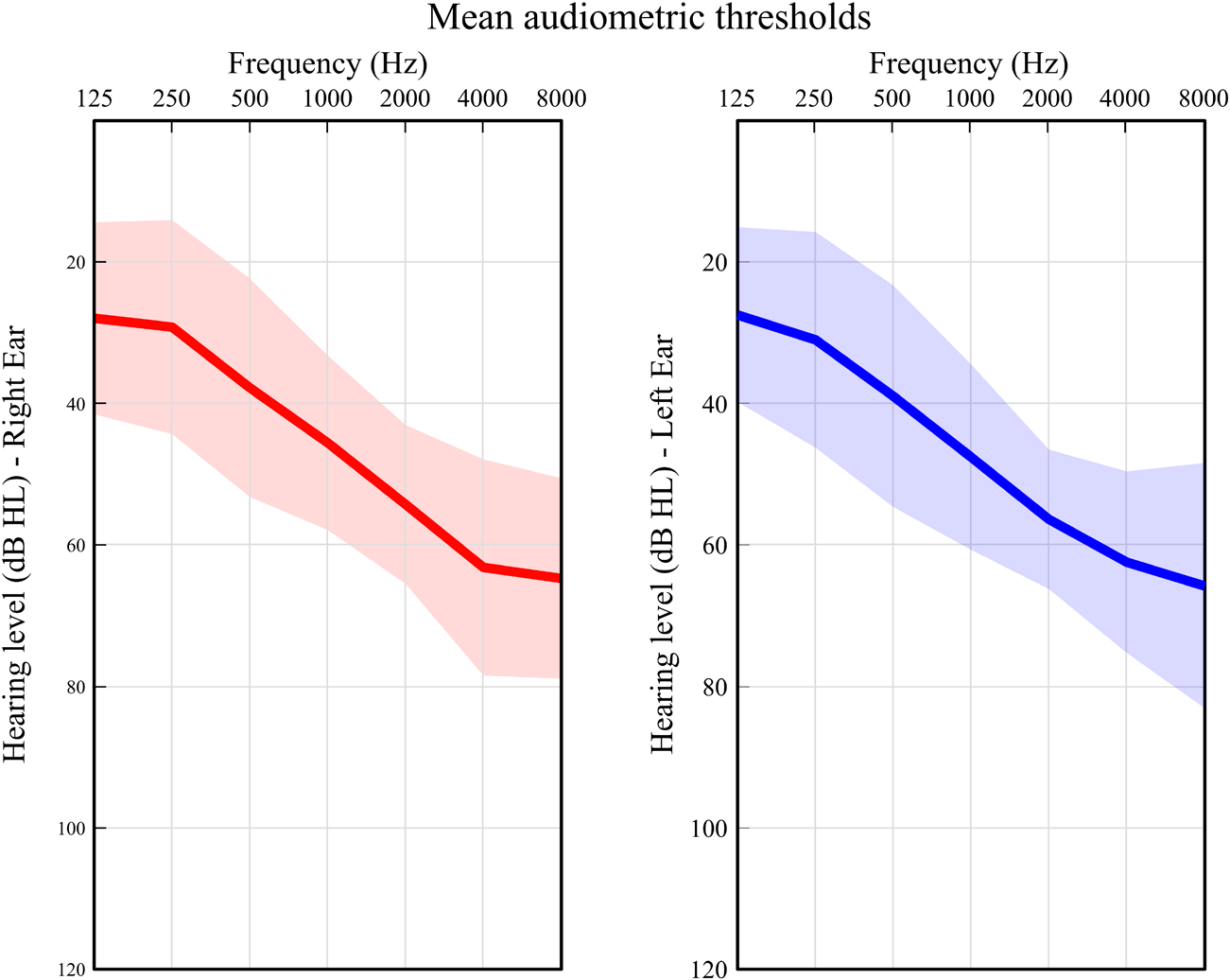
Mean hearing thresholds and standard deviations.

Participants had relatively uniform degrees of HL with a mean 4F-PTA of 50.2dB HL (SD = 8.9) in the right ear and 51.3 dB HL (SD = 8.7) in the left ear (see Fig. 1 for average thresholds per frequency). During testing, participants wore Oticon OPN S1 miniRITE hearing aids programmed based on their most recent audiogram using the Oticon Genie 2 fitting software. Noise reduction, directionality, and button functionality were turned off. A first fit was used, no adjustments were made. The hearing aid amplification was not verified by real-ear measurements. This was similar to the approach of the fitting of their personal hearing aids. Participants did not use their own hearing aids during testing to reduce variability associated with different signal processing algorithms and hearing aid settings. All but one participant used power domes, and one individual wore standard domes because they found the power domes uncomfortable. Participants were not paid to participate in this experiment. Approval for this study was granted by the Research Ethics Committees of the Capital Region of Denmark.

A priori data simulations—as performed through the Simr package (Green & Macleod 2016) in R programming language (R Core Team 2020)—were used to estimate the required sample size to detect changes in pupil dilations related to copresence and task difficulty (alpha of 0.05). Specifically, the simulations used parameter estimates (β) found by Pielage et al. (2021), who manipulated SiN difficulty by means of intelligibility and copresence by means of having two participants take turns on a SiN task. These parameter estimates were rounded down in order to account for smaller effect sizes (intercept: 0.22, easy to difficult conditions: −0.04, alone to copresence conditions: 0.02). The simulations revealed that 13 participants would have been sufficient to achieve 80% power. However, power calculations related to the cardiovascular measures described in Plain et al. (2021) indicated that 26 participants were required. In total, 29 participants were included to account for potential data losses.

### Procedure

Following informed consent, participants completed a self-efficacy questionnaire (described below) and were then fitted with the Oticon OPN hearing aids. Next, participants were seated in a sound treated room with an ambient illumination of 200 lx, behind a desk on which an eye-tracker was mounted. Electrodes were placed on the participants’ neck and side to record cardiovascular data (reported elsewhere, see: Plain et al. 2021). Every participant then completed seven SiN blocks consisting of 20 sentences each; one practice block, two adaptive blocks used to individually estimate speech reception thresholds (SRTs) (Plomp & Mimpen 1979) at 50% and 80% sentence intelligibility, and four experimental blocks. The practice and adaptive blocks were always performed first and without observers copresent. Next, participants completed the experimental blocks. These began with a five-minute clip of a neutral video (aerial shots of Scotland), during which baseline cardiovascular measurements were taken. After an experimental block had concluded, participants were asked to complete several subjective ratings (see paragraph subjective ratings below). There was a 10-minute break after two experimental blocks during which participants could stand up and move around. To avoid copresence effects caused by the experimenter, the task was controlled from an adjacent room. The participant and experimenter could not see each other, but communication was possible through an intercom system.

### Task and Stimuli

**Fig. 2.**
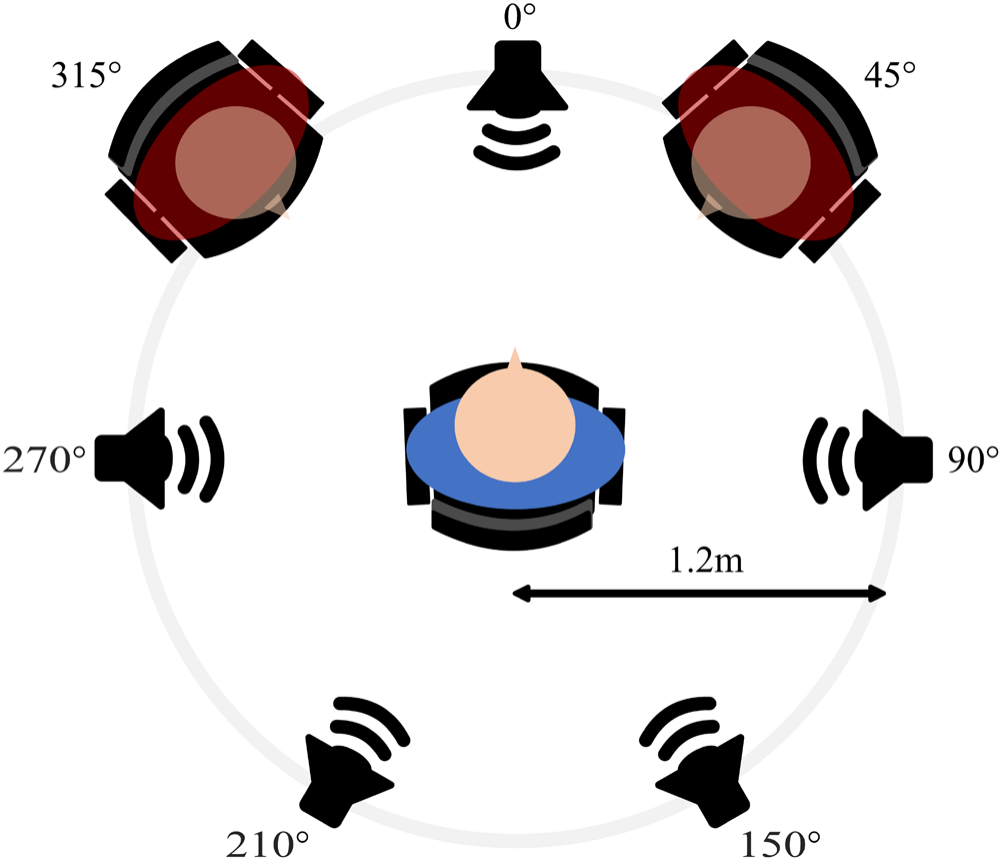
Schematic display of the experimental setup. The participant (blue) listened to target sentences played from the loudspeaker in front of them. Masker recordings were played from all other loudspeakers. Observers (red) were seated diagonally opposed to the participant.

For the SiN task, participants repeated back sentences uttered by a female talker. Target sentences, taken from the Danish HINT (Nielsen & Dau 2011), were played from a loudspeaker in front of the participant (see Fig. 2). The sentences consisted of five key words each, and had an average duration of 1.5 seconds (range 1.2–1.9 seconds). An example sentence is “Bussen kan ikke komme frem,” which translates to “The bus cannot move forward.” Target sentences were masked by four (2 males and 2 females) continuous speech recordings, consisting of random cuts from newspaper readings (full recordings were each 10 minutes long) from which all pauses longer than 50ms were removed (Wendt et al. 2018). The masker recordings were spectrally shaped to have a similar long-term average frequency spectrum as the target sentences. Each masker recording was played from a separate loudspeaker located 1.2 m away from the participant at either 90, 150, 210, or 270 degrees azimuth (Fig. 2) (Wendt et al. 2018). Each trial started and ended with a period of 3 seconds during which only the masker was playing, with the presentation of the masked sentence in between. The 3 seconds of masker following the sentence provided time to capture the full pupil response. The participant repeated the target sentences once the masker had stopped playing. Trials were advanced manually by the experimenter who left a minimum interval of three seconds between offset of the repetition and the onset of the next trial.

### Practice and Adaptive Blocks

Prior to data collection, participants completed two adaptive blocks during which signal-to-noise ratios (SNRs) were altered in accordance with an adaptive procedure that estimated the participant’s SRTs at 50% and 80% sentence correct (Kaernbach 1991). These adaptive protocols were based on the HINT Pro software (House Ear Institute 2005). To familiarize participants with the task, the adaptive blocks were preceded by a practice block, which was similar to the adaptive block to estimate the SRT at 50% sentence intelligibility. The 50% procedure was used for practice so participants were aware of how difficult the experimental task could be. SNRs were altered by varying the sound level of the target sentences, while the sound level produced by the four masker recordings combined remained fixed at 70 dB SPL. The practice block, as well as the adaptive block for 50% intelligibility, started at an SNR of +6 dB using step sizes of 4 dB up and 4 dB down. The 80% intelligibility procedure started at an SNR of +10 dB using step sizes of 6.4 dB up and 1.6 dB down. After the fifth trial (that used an SNR equal to the average SNR of the first four trials), step sizes were halved.

### Experimental Blocks

During the experimental blocks, SNRs were fixed to coincide with either the acquired SRT at 50% sentence intelligibility (difficult block) or at 80% sentence intelligibility (easy block), these blocks were thus not adaptive. This resulted in a more consistent task performance across participants, as opposed to using the same SNR for all participants. While this could also be achieved by using adaptive procedures during the experimental blocks, the current method was preferred to minimize between-trial variance in task difficulty. SNR was varied by changing the sound level of the target sentences, while the masker level remained fixed. Copresence was manipulated by having participants perform the SiN task either alone or with two observers copresent (see also: Plain et al. 2021); that is, a fully crossed 2 (copresence) × 2 (difficulty) within-subjects design.

The observers were seated 1.2 m from the participant at 45 and 315 degrees azimuth (see Fig. 2). This arrangement was used so that participants could see the observers in the periphery of their vision, ensuring that they were aware of their presence throughout testing. Participants and observers were unfamiliar with one another; however, they were of similar ages to simulate the copresence of (plausible) social peers. All observers were hearing aid users and none were experiment participants themselves. Observers were instructed not to communicate with the participant but to watch the participant during testing while maintaining a “positive and nonthreatening demeanor.” Observers and participants were told to imagine that they were in a bar or restaurant having a conversation. To ensure observers were engaged during testing they were asked to form an opinion about the participant’s listening performance and to write a short summary of their observations after each block. No guidelines were supplied for this, as there was no intention to use these data. The order of experimental blocks was counter-balanced over participants, with the restriction that the two alone blocks were always performed sequentially. The same was true for the two copresence blocks.

Performance during the experimental blocks was assessed by means of sentence correct scores on a trial level. Sentence correct scores could either be 0 (incorrect repetition) or 1 (correct repetition). To coincide with the pupil data analysis (described below), the first four trials in a block were excluded from analysis.

### Pupil Size

Pupil size was recorded continuously during each experimental block using a Tobii Spectrum eye-tracker (Tobii AB) set to sample at 600 Hz. To avoid gaze artifacts on the pupil data, participants were asked to fix their gaze on the loudspeaker in front of them whenever sound was playing. At the onset of every target sentence, a trigger was sent to the eye-tracker so that the data could be separated into trials. Data were taken from the right eye only, as there are some indications that it better reflects cognitive load than the left (Liu et al. 2017; Wahn et al. 2017). Segments of data representing a trial (pupil traces) were cut to be 7.2 seconds long, consisting of 3 seconds before target sentence onset, at least 1.2 seconds of target sentence presentation (length of shortest sentence) and three additional seconds to capture the full pupil response. Only the traces corresponding to trials 5 to 20 from each block were used for analysis. The first four sentences were excluded to allow the participant time to adjust to the test condition (Winn et al. 2018).

Raw pupil traces were cleaned to remove blinks and other artifacts from the recordings. Some traces had very brief segments of missing data of unknown origin, which were too short to be caused by blinks. The occurrence of these segments was highly variable between participants, but on average they occurred 13.5 times per sentence (SD = 44.5; median = 6) with an average length of 2 samples (3 ms) and a standard deviation of 1.1 samples (1.8 ms). On average, they occupied less than 1% of the full trace (SD = 2%). The high sampling rate preserved clear trends in the data in all cases. Missing samples of these segments were replaced through linear interpolation from the last nonmissing sample to the third consecutive nonmissing sample after the segment (to link segments in close proximity). Any missing segment longer than 15 samples (25 ms) was regarded as a blink and not interpolated at this stage.

After the small missing data segments had been interpolated, traces which still had more than 50% missing samples were excluded from further analyses. This is a lenient threshold compared to previous work (Ohlenforst et al. 2018; Winn et al. 2018). However, all traces adhering to this criterion were found to reliably show the overall pupil size morphology. On average, sentences had 13.8% missing data caused by blinks (SD = 12.6%). If more than 5 of the 16 traces from a block did not make the quality threshold, the entire block was excluded from further analyses. For five participants, this occurred in all experimental conditions. One participant had this occur in two conditions and for one participant only a single conditions was excluded. When a trace passed the quality threshold, any remaining missing samples were considered to be blinks, which were dealt with as follows. First, the 50 samples (83 ms) before the first missing sample of a blink and all samples between the last missing sample of the blink and the 80th consecutive nonmissing sample (133 ms) were removed to account for artifacts of the eye closing and opening (Koelewijn et al. 2012). Next, to reduce high-frequency noise, the trace was smoothed using a moving average filter with a width of 51 samples that skipped over missing data. Finally, the missing samples were replaced through linear interpolation. This preprocessing pipeline differed from that used in similar studies, in which smoothing is applied after interpolation (Winn et al. 2018). This was done to avoid interpolation between noisy values.

After preprocessing, pupil traces were baseline corrected by subtracting BPS from all values within the trace. Similar to previous studies (Ohlenforst et al. 2018; Koelewijn et al. 2021), BPS was defined as the average pupil size during the last second of the masker before target sentence onset. Next, all traces belonging to a condition were averaged into one mean trace. From this mean trace, the task-evoked pupil response (TEPR) was defined as the portion of the trace between target sentence onset and masker offset. Finally, PPD was defined as the maximum value within the TEPR and MPD was the average of all values in the TEPR.

### Subjective Ratings

After each experimental block, participants rated the (1) effort they invested during the task using a question adapted from Zekveld et al. (2010); (2) likelihood of giving up on listening and (3) likelihood of trying to improve/change their listening environment if the test would have been a real-life scenario, using questions adapted from Picou and Ricketts (2014, 2018) and (4) stress experienced during the task using a question adapted from Mackersie & Cones (2011). Participants responded on a visual analogue scale ranging from 0 to 10 with tick marks at every one-decimal step.

To assess whether listening-related self-efficacy might have been related to copresence, participants completed a questionnaire after the experiment, which was adapted from the Listening Self-Efficacy Questionnaire (LSEQ) (Smith et al. 2011). Participants rated 10 listening scenarios (e.g., talking to a person you know well, such as a close friend or family member) for their expected listening performance (as per the LSEQ) and their confidence for managing the scenario as used in the Self-Efficacy for Situational Communication Management Questionnaire (Jennings et al. 2014). Participants were asked to make ratings assuming they were wearing their own hearing aids. This was done to get an idea of the participants’ self-efficacy during normal daily-life situations. However, as a result, the questionnaire was not necessarily reflective of self-efficacy experienced during the experiment.

### Statistical Analyses

The lme4 package (Bates et al. 2015) in R (R Core Team 2020) was used to model performance, pupil, and subjective measures with mixed-effect models. As performance was measured by means of trial-level sentence correct scores, a binomial mixed-effect model was fitted to the performance data. Starting with a null-model that only included the intercept and random effects structure, a step-up method was used to assess if the addition of task difficulty, copresence, and hearing status improved the amount of variance explained by the model. The random effects structure included the by-participant intercept only. Even though it has been recommended to include all possible random effects (intercepts and slopes) (Barr et al. 2013), the sample size was not large enough to support such complex models, resulting in overfitting and convergence problems.

For the pupil and subjective rating data, a different approach was used. First, an initial mixed-effect model was fitted to the condition-level data which included fixed factors for both experimental manipulations and their interaction (lme4 notation: y ~ difficulty*copresence + (1|participants)). After fitting this model, fixed factor significance was assessed using a Type-III analysis of variance (ANOVA) with Satterthwaite’s estimation of degrees of freedom (Satterthwaite 1946). There is some debate about the usefulness of F-tests in linear mixed-effect models, since there is no clear consensus about the degrees of freedom that should be used (Luke 2017). Therefore, parameter estimates (β) and their 95% confidence intervals (CIs) are reported as well. To check if age, PTA, and self-efficacy (together with relevant interactions) were related to the pupil data, they were added to the models using a step-up method. In the case of self-efficacy, a single value was calculated by taking the mean of the “confidence in managing the scenario” ratings (Jennings et al. 2014). If one or more steps resulted in significantly more explained variance compared to initial model, they would be included in the model and fixed factor significance would be reassessed using an F-test.

## RESULTS

### Final Dataset

Data from five participants were excluded as the number of pupil traces passing the quality threshold was insufficient in all four experimental conditions. Subthreshold quality of pupil data was often due to the eye-tracking system failing to acquire a good signal, likely caused by upper eyelids that partly obscured the pupil. In addition, there were two participants from whom the data of individual conditions were excluded. For one participant, the alone-easy block was omitted, and for another participant both easy blocks were omitted because of poor data quality. Mixed-effect models are able to deal with missing data, thus the remaining data from these participants were included in the analyses.

### Performance

**Fig. 3.**
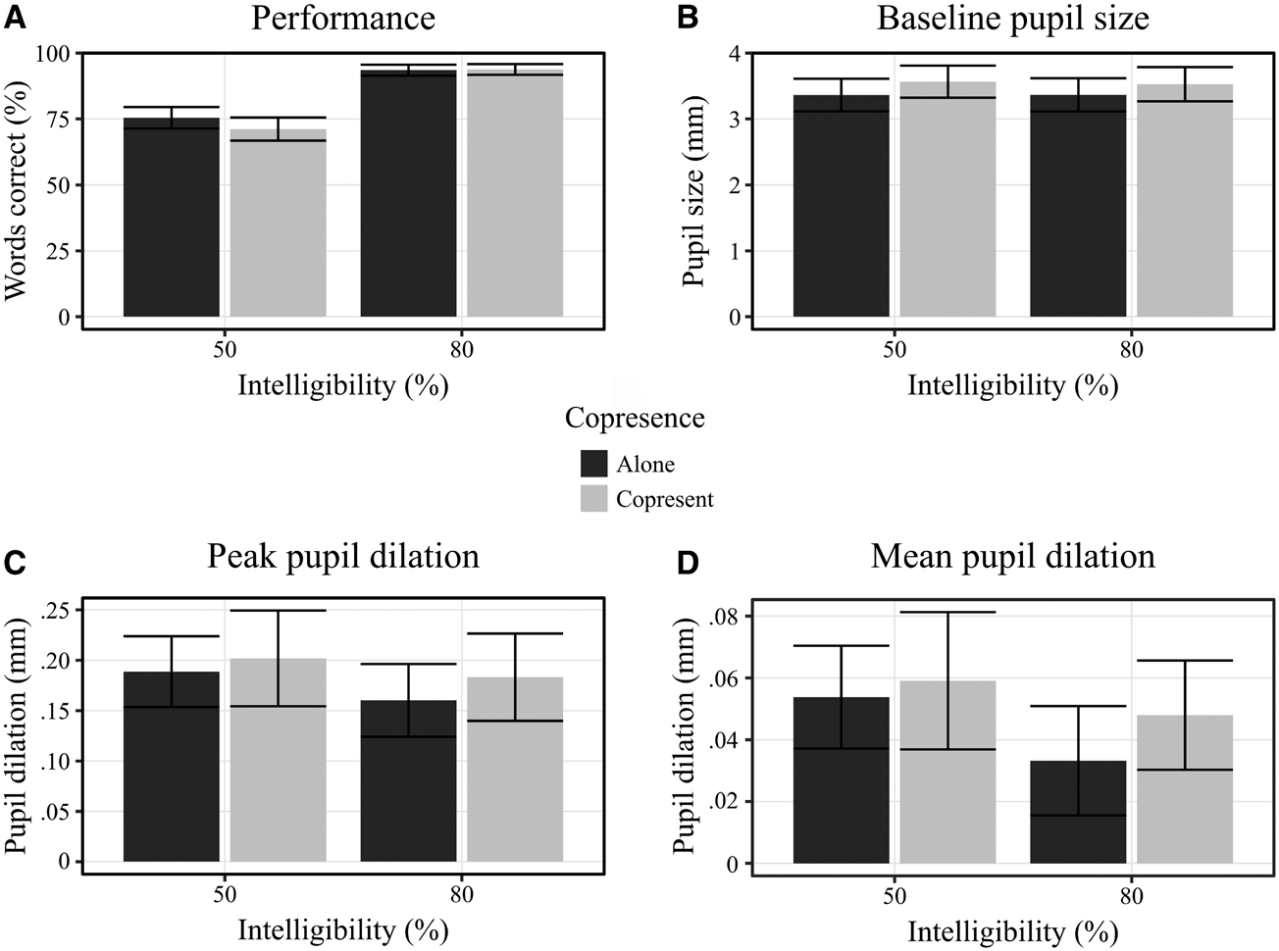
Plots of performance and pupil measures, together with standard errors. Subplots correspond to (A) the percentage of sentences repeated correctly, (B) mean baseline pupil size, (C) peak pupil dilation, and (D) mean pupil dilation. Asterisks correspond to significant effects as found by the F-test.

Because the SNRs used in the experimental blocks were based on the performance of the participant during the adaptive SRT blocks, they varied between participants. These SNRs ranged from +2.2 to +10.9 dB (average: +5.4 dB) for the difficult conditions and from +4.9 to +18.6 dB (average: +10.2 dB) for the easy conditions. The differences in SNR between conditions ranged from +1.8 to +8 dB between participants. Given these SNRs, Figure 3A shows the percentage of sentences participants correctly repeated during the experimental blocks. Results from the step-up method which added fixed factors to the mixed-effect model predicting performance can be found in Table 1. The table shows that adding task difficulty and 4F-PTA as main effects significantly improved model fit.

**TABLE 1.** Results of adding fixed factors to the binomial model predicting sentence correct scores. Bold lines indicate steps that significantly improved model fitness

	Fixed Factors	Χ^2^	Df	*p*
Step 1	Task Difficulty	195.05	1	<0.01
Step 2	Task Difficulty + Copresence	3.01	1	0.08
Step 3	Task Difficulty + Task Difficulty × Copresence	3.20	2	0.20
Step 4	Task Difficulty + PTA	7.44	1	<0.01
Step 5	Task Difficulty + PTA + Task Difficulty × PTA	0.25	1	0.62
Step 6	Task Difficulty + PTA + Copresence × PTA	3.58	1	0.06

PTA indicates pure-tone average.

published online ahead of print April 4, 2023.

We took the exponential of the observed beta values and their CIs to interpret them as odds ratios. The final model predicted that an increase of N dB 4F-PTA resulted in a decrease of 0.976^N^ in the odds of repeating a sentence correctly. When 4F-PTA was constant, the odds of repeating a sentence correctly in the easy condition were greater than those in the difficult condition by a ratio of 5.226. This suggests that performance was better for easy conditions compared to difficult ones, which is also shown in Figure 3A. As SNRs in the easy condition were fixed to participants’ SRT at 80% sentence correct level, the ratio of correct to incorrect sentence repetitions in easy conditions should have been approximately 4 (8/2). The same logic applies to difficult conditions with a ratio of approximately 1 (5/5). As such, theoretically we expected the odds ratio of correct responses for the easy compared to the difficult condition to be approximately 4 (4/1). This means that the results suggest that performance differences between the conditions were slightly greater than anticipated.

### Pupil Data

**Fig. 4.**
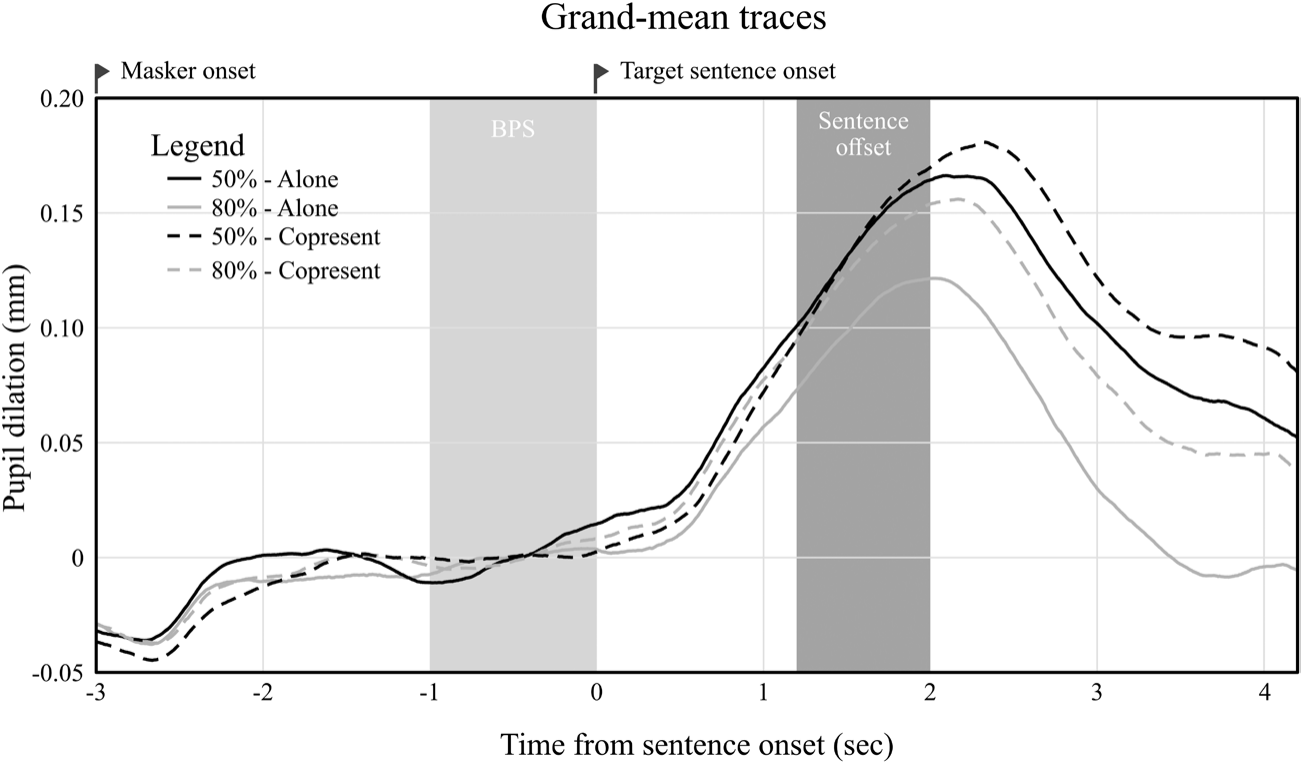
Grand mean pupil traces. At −3 seconds the masker noise started playing. The gray box ranging from minus one to zero seconds indicates the period during which baseline pupil size (BPS) was measured. Zero seconds marks the start of the target sentence. The dark gray box indicates the range of target sentence offsets (1.2 to 1.9 seconds). The *x* axis ends after the length of the shortest sentence +3 seconds of masker.

Mean pupil traces for the valid data were averaged over participants, resulting in one grand mean trace per condition. These traces are plotted in Figure 4. The plots show well pronounced TEPRs with their peak occurring roughly 2 seconds after target sentence onset. Trends for both task difficulty and copresence can be seen where the difficult and copresence conditions were associated with relatively larger pupil dilations.

### Baseline Pupil Size

**Fig. 5.**
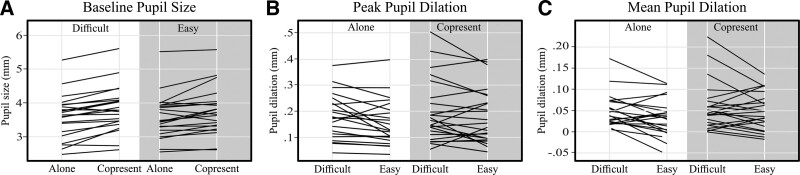
Individual-level results of (A) BPS (alone vs copresent), (B) PPD (easy vs difficult), and (C) MPD (easy vs difficult). BPS indicates baseline pupil size; MPD, mean pupil dilation; PPD, peak pupil dilation.

BPS values of all included trials in a block were averaged to create one mean BPS score per condition, per participant. Mean BPS values are plotted in Figure 3B. After fitting the initial model, no obvious deviations from the assumptions of homoscedasticity and linearity were detected through visual inspection of residual plots. F-statistics revealed a significant association between copresence (*F*(1,68) = 46.50, *p* < 0.01) and BPS, but not between difficulty (*F*(1,68) = 2.00, *p* = 0.16) and BPS. Parameter estimates and CIs can be found in Table 2. The parameter estimate for copresence seems to suggest that mean BPS was higher for copresence conditions (β = 0.219 mm), compared to alone conditions. This relationship is also visible in Figure 3B, in which it can be seen that copresence was associated with higher BPS in both the easy and difficult conditions. The narrow CIs of the copresence effect seem to suggest that this relationship was relatively consistent. This can also be seen in Figure 5A. Only a few participants showed a reverse association between copresence and BPS. The CIs for the difficulty estimate are wide and encompass zero, providing insufficient evidence supporting a relationship between task difficulty and BPS. No significant interaction between difficulty and copresence was found either (*F*(1,68) = 0.84, *p* = 0.36), which is supported by a parameter estimate close to zero and unfavorable CIs. Adding age (main effect), 4F-PTA (main effect and interaction with difficulty) and self-efficacy (main effect and interaction with copresence) as fixed factors did not yield significant improvements in variance explained by the model (see Table 3).

**TABLE 2.** Parameter estimates (β) and 95% CIs of BPS, PPD, and MPD

	BPS				PPD			
	β	CI (95%)			β	CI (95%)		
Intercept	+3.577	+3.324	to	+3.829	+0.190	+0.150	to	+0.229
Difficulty (difficult -> easy)	−0.014	−0.094	to	+0.065	**-0.028**	−**0.055**	**to**	**+0.000**
Copresence (alone -> copresent)	**+0.219**	**+0.141**	**to**	**+0.296**	+0.012	−0.015	to	+0.039
Intelligibility × Copresence	−0.052	−0.163	to	+0.059	+0.011	−0.027	to	+0.050
	MPD							
	Β	CI (95%)						
Intercept	+0.054	+0.036	to	+0.072				
Difficulty (difficult -> easy)	−**0.019**	−**0.034**	**to**	−**0.005**				
Copresence (alone -> copresent)	+0.005	−0.009	to	+0.019				
Intelligibility × Copresence	+0.009	−0.011	to	+0.029				

Parameter estimates for difficulty use the difficult conditions as baseline and represent the change going from the difficult condition to the easy condition. The parameter estimates for copresence use the alone condition as baseline and represent the change going from alone to copresent. Bold parameter estimates and CIs correspond to significant effects as found by the F-test.

BPS indicates baseline pupil size; CI, confidence interval; MPD, mean pupil dilation; PPD, peak pupil dilation.

**TABLE 3.** Results of step-up method of adding age, PTA. and self-efficacy to the model fitted to pupil data

	Fixed factors	BPS	PPD	MPD
Step 1	Age	χ^2^(1) = 0.04, *P* = 0.85	χ^2^(1) = 0.05, *P* = 0.82	χ^2^(1) = 0.22, *P* = 0.64
Step 2	PTA + PTA × Difficulty	χ^2^(2) = 0.01, *P* = 0.99	χ^2^(2) = 3.39, *P* = 0.18	χ^2^(2) = 3.23, *P* = 0.20
Step 3	Self-Efficacy + Self-Efficacy × Copresence	χ^2^(2) = 1.43, *P* = 0.49	χ^2^(2) = 1.64, *P* = 0.44	χ^2^(2) = 2.24, *P* = 0.33

Unlisted interactions were not considered to be of interest and therefore not tested.

BPS indicates baseline pupil size; CI, confidence interval; MPD, mean pupil dilation; PTA, pure-tone average; PPD, peak pupil dilation.

### Peak Pupil Dilation

Average PPD values are plotted in Figure 3C. After fitting the initial model, residual plots indicated no substantial deviation from the assumptions. F-statistics suggested a relationship between PPD and difficulty (*F*(1,69) = 4.70, *p* = 0.03), but not between PPD and copresence (*F*(1,68) =3.44, *p* = 0.07). Nor did the F-statistics suggest an interaction (*F*(1,68) = 0.37, *p* = 0.55). While Figures 3C and 4 indeed seem to suggest that PPD and task difficulty were related, note that the CIs of the parameter estimate encompass zero. This suggests a weak relationship, which should be interpreted with care. The individual effects plotted in Figure 5B show that there is a lot of variability between participants. Adding age (main effect), PTA (main effect and interaction with difficulty), and self-efficacy (main effect and interaction with copresence) as fixed factors did not yield significant improvements in variance explained by the model (see Table 3).

### Mean Pupil Dilation

MPD values are shown in Figure 3D. No apparent deviation from the assumptions of homoscedasticity and linearity were found after fitting the initial model. F-statistics revealed that MPD was significantly related to task difficulty (*F*(1,69) = 8.26, *p* < 0.01) but not copresence (*F*(1,68) = 3.67, *p* = 0.06). No interaction was found (*F*(1,68) = 0.78, *p* = 0.38). Parameter estimates (Table 2) revealed that MPD was lower during easier blocks compared to the more difficult ones (β = −0.019 mm), which is supported by the plotted data. Wide CIs suggest high variance between participants, which is confirmed by the plotted individual effects in Figure 5C. Although the F-statistics for the relationship between MPD and copresence approached significance, the parameter estimate and CIs do not support that there was a consistent relationship. Adding age (main effect), PTA (main effect and interaction with difficulty), and self-efficacy (main effect and interaction with copresence) as fixed factors did not yield significant improvements in variance explained by the model (see Table 3).

### Cluster-based Permutation Analysis

Despite that copresence was not found to be associated with PPD or MPD, Figure 4 shows a trend whereby copresence did seem to be related to pupil size in the second half of the pupil response. PPD and MPD condense the TEPRs to a singular data point, thus all temporal information is lost. Any relationship between pupil size and the manipulations during specific intervals would therefore go unnoticed. An exploratory cluster-based permutation analysis, as inspired by (Maris & Oostenveld 2007), was applied to analyze the TEPRs in further detail. Since this method utilized a repeated-measures ANOVA, which does not allow for missing data, only participants with a complete data set were included (n = 22). The cluster-based permutation analysis applied a repeated-measures ANOVA to each time point in the TEPRs (target sentence onset to masker offset). If two or more adjacent points in time were significantly associated with the same independent variable, they were grouped into a cluster. For example, if 20 adjacent time points showed a significant relationship between pupil size and the copresence manipulation, this was considered as one cluster. This was done separately for both main effects (difficulty and copresence) as well as their interaction. Because all three effects of the 2 × 2 design were considered, a Bonferroni corrected alpha of 0.017 (0.05/3) was used to assess significance at each time point.

A permutation method was used to assess if the found clusters had any statistical meaning. For each cluster, all *F*-values (one per time point) were summed to calculate the sum of *F* for that cluster (∑*F*). Next, the labels of all conditions were randomly assigned to the data of each participant, which was repeated for 2000 permutations. From each of these permutations, analogous to the originally labeled data, clusters of neighboring significant values had their summed *F*-value calculated (∑*F*_perm_). The maximum ∑*F*_perm_ of each permutation and each comparison was extracted, resulting in one permutation distribution of ∑*F*_perm_ per comparison. These distributions resemble chance levels of finding certain ∑*F*. From these distributions, critical thresholds (∑*F*_crit_) were estimated at the 95th percentile. A cluster of the original labeled data was considered significant if its ∑*F* exceeded ∑*F*_crit_ for that comparison, signaling that it was statistically unlikely. The *p* value was defined as the proportions of permutations where ∑*F*_perm_ exceeded ∑*F*.

Results showed a significant cluster for difficulty starting after 2.23 seconds after target sentence onset which lasted to the end of the trace (*F*_perm_ < 0.01) and for copresence starting after 2.48 seconds after target sentence onset, also lasting until the end of the trace (*F*_perm_ < 0.01), suggesting a significant relationship between pupil size and both copresence and task difficulty during the second half of the TEPR. There was no significant cluster for the interaction term.

### Subjective Ratings

Scores on the subjective rating scales can be found in Table 4. Similar to the analyses of the pupil data, a model including difficulty, copresence and their interaction was fitted to the data from each subjective rating scale. All four ratings were found to be significantly associated with task difficulty (effort: *F*(1,69) = 57.42, *p* < 0.01, change situation: *F*(1,69) = 39.74, *p* < 0.01, give up: *F*(1,68) = 29.08, *p* < 0.01 and stress: *F*(1,70) = 42.19, *p* < 0.01), but not copresence (effort: *F*(1,68) = 0.74, *p* = 0.39, change situation: *F*(1,69) = 0.58, *p* = 0.45, give up: *F*(1,66) = 0.32, *p* = 0.57, and stress: *F*(1,70) = 3.43, *p* = 0.07) or their interaction (effort: *F*(1,68) = 0.37, *p* = 0.655, change situation: *F*(1,69) = 0.31, *p* = 0.58, give up: *F*(1,66) = 0.02, *p* = 0.89, and stress: *F*(1,70) = 0.05, *p* = 0.82). Parameter estimates (Table 5) indicate that difficult blocks were associated with higher scores for all subjective ratings compared to easier blocks. The parameter estimates and CIs for copresence and the interaction terms are not suggestive of any relationships.

**TABLE 4.** Mean subjective rating scores and standard deviations (SDs)

		Alone		Copresent	
Difficulty	Rating	Mean	SD	Mean	SD
Difficult	Effort	6.40	1.71	6.85	1.53
	Change situation	7.38	2.31	7.35	1.91
	Give up	3.82	2.60	4.10	2.88
	Stress	4.90	2.44	5.46	2.50
Easy	Effort	4.30	2.11	4.36	1.91
	Change situation	5.30	2.95	4.82	3.03
	Give up	1.91	1.51	2.17	2.11
	Stress	3.05	1.07	3.54	2.26

**TABLE 5.** Parameter estimates (β) and 95%CIs of the subjective ratings

	Effort				Change situation			
	β	CI (95%)			β	CI (95%)		
(Intercept)	+6.397	+5.659	to	+7.135	+7.428	+6.411	to	+8.445
Difficulty (difficult -> easy)	**−2.170**	**−3.042**	**to**	**−1.299**	**−2.101**	**−3.123**	**to**	**−1.078**
Copresence (alone -> copresent)	+0.453	**−**0.395	to	+1.302	**−**0.074	**−**1.067	to	+0.920
Intelligibility × Copresence	**−**0.376	**−**1.589	to	+0.838	**−**0.403	**−**1.824	to	+1.018
	Give up				Stress			
	Β	CI (95%)			β	CI (95%)		
(Intercept)	+3.934	+2.989	to	+4.880	+4.907	+4.003	to	+5.810
Difficulty (difficult -> easy)	**−2.136**	**−3.217**	**to**	**−1.055**	**−2.064**	**−2.928**	**to**	**−1.201**
Copresence (alone -> copresent)	+0.161	**−**0.890	to	+1.213	+0.495	**−**0.344	to	+1.334
Intelligibility × Copresence	+0.110	**−**1.394	to	+1.614	+0.138	**−**1.056	to	+1.333

Parameter estimates for difficulty use the difficult conditions as baseline and represent the change going from the difficult condition to the easy condition. The parameter estimates for copresence use the alone condition as baseline and represent the change going from alone to copresent. Bold parameter estimates and CIs correspond to significant effects as found by the F-test.

CI indicates confidence interval.

## DISCUSSION

This study assessed if there was a relationship between copresence and SiN task performance or effort in persons with HL. Copresence was manipulated by having participants perform a SiN task both alone and while two observers were copresent. SiN performance, task-related effort and arousal (as measured through pupillometry), and subjective ratings were examined. To determine whether copresence interacted with the demands imposed by the listening task, task difficulty was manipulated by testing at two SNRs, corresponding to 50% and 80% sentence intelligibility (referred to as difficult and easy, respectively).

We had hypothesized that BPS would remain stable across conditions; however, this was not the case. Instead, the copresence conditions were associated with larger BPSs compared to the alone conditions. This was not due to the influence of the pupil response of the previous trial because the pupil was given time between trials to return to baseline. As such, the increase in BPS suggests an increase in arousal (Granholm & Steinhauer 2004; Wang et al. 2018; Zekveld et al. 2018). Indeed arousal has been found to be influenced by copresence before (Bond & Titus 1983).

The relationship between copresence and BPS can be explained by social self-preservation theory, which predicts greater arousal when there is the possibility for the self to be judged negatively by others (Gruenewald et al. 2004, 2007; Bosch et al. 2009). The presence of the observers in this study could have led participants to worry they were being judged negatively, perhaps because they had low confidence in their ability to do the task due to their HL (Jennings et al. 2014). As a result, participants could have been more anxious about the task (Lempert et al. 2015) or could have premobilized effort in an attempt to overcome the upcoming hearing challenges (Koelewijn et al. 2014; Pichora-Fuller et al. 2016). However, it might also be that the observers elicited discomfort in participants that was unrelated to the task, but because the experimental setup was somewhat unnatural. It is noteworthy that while greater arousal might be experienced as stress, subjective ratings of stress were not found to be associated with the copresence manipulation.

A few participants showed a reversed relationship between copresence and BPS, where their BPS was lower during copresence conditions, compared to the alone conditions. This was not associated with age, PTA or self-efficacy. It might be that participants whose BPS was lower during the copresent conditions perceived the observers to be supportive, whereas those with a higher BPS under the copresent condition perceived the observers to be threatening. This highlights the need to consider individual differences and potential social ties between individuals when using similar research designs.

In this study, copresence was not associated with a change in task performance, which differs from earlier research on copresence (Bond & Titus 1983). In social facilitation and inhibition literature, it has been hypothesized that copresence affects performance through increased motivation and/or effort (McFall et al. 2009; Belletier et al. 2019). Possibly, SiN task performance is not sensitive to copresence manipulations because it is mainly determined by the acoustical quality of the signal, less so by motivation and/or effort. In contrast to listening performance, listening comprehension has been found to be related to copresence (Beatty 1980), suggesting that other listening-related outcomes might be more strongly associated with copresence during listening.

Copresence was not associated with a change in PPD or MPD. This could be due to several reasons: (1) the increase in BPS obscured the pupil dilation response; (2) copresence is not related to effort during SiN tasks; and/or (3) the way copresence relates to PPD and MPD varied a lot between participants and too few participants were included to capture a statistically meaningful relationship. Findings by (Reilly et al. 2019) suggest that the former explanation (1) is unlikely, as they found that the dilation response scaled linearly with effort, regardless of BPS. In line with explanation 3, there is a lot of variability between participants in how copresence was related to PPD/MPD. This might be because some participants perceived the observers as supportive while others found them intimidating. But, the variability could also reflect random noise. Therefore, to better differentiate between the latter two explanations (2 and 3), more research is required.

A post-hoc cluster-based permutation analysis revealed that the second part of the TEPR was sensitive to differences between copresence conditions. A recent study has pointed out that the time course of the TEPR can be dissected into multiple components reflecting different processes related to SiN tasks (such as active listening, sentence processing and repeating back sentences) (Książek et al. 2021). It could be that copresence was related to some, but not all of these processes. For example, as the difference in pupil size between copresence conditions was only observed at the second half of the TEPR, it could be that copresence is only associated with an increase in effort in the process of preparing to respond. However, to make more definitive conclusions, future studies should be designed specifically around researching this.

None of the subjective ratings were found to be related to copresence. This might be because participants did not experience differences in subjective effort between copresence conditions or because the questions did not address the relevant topics.

While listening in the presence of others is a part of many daily-life listening situations, we acknowledge that the specific situation used here was not an entirely natural situation. Nonetheless, because this study showed that copresence was related to arousal in persons with HL, it is worth conducting further research on the topic using more realistic listening situations. Such studies could further advance our knowledge about factors that hinder or promote listening. Furthermore, although no evidence was found that copresence was associated with listening performance decrements, clinicians may want to be aware that their presence during hearing assessments could influence arousal and task-related effort of persons with HL.

It should be noted that the study had several limitations. Notably, it seems that the manipulation of difficulty did not fully achieve the desired goal of equalizing task difficulty between participants. This is evident from the finding that a higher PTA was associated with decreased performance, suggesting that the task was more difficult for those with greater degrees of HL. This might explain the high variability in the relationship between task difficulty and PPD as well as MPD (Zekveld et al. 2018). Another limitation involves the high-frequency noise and small segments of missing data that affected the pupil data quality of several participants. While preprocessing yielded good results for these participants, the noisy raw data could still have biased the outcome measures. For example, a possibly weaker relationship between copresence and PPD might have been undetected due to the noisy signal. Finally, because people with normal hearing did not participate, it is not possible to determine whether the effects here are specific to persons with HL. Future studies should include both persons with HL and age-matched persons with normal hearing in order to make more definitive statements about potential differences in how copresence is associated with the studied parameters in the two groups.

## CONCLUSION

This study provided evidence that global arousal, as reflected in BPS, is associated with copresence among persons with HL. This could be related to task-related anxiety, premobilization of effort and/or discomfort in response to the observers’ presence. Effort related to listening was not found to be related to copresence. However, copresence might have been associated with increased effort related to other processes involved in a SiN task, such as preparing to respond. Because the data are somewhat inconclusive and because copresence might manifest differently in daily-life, further research examining copresence is required—perhaps using different testing paradigms, larger samples sizes and with more diverse participants, such as people without HL.

## ACKNOWLEDGMENTS

This project has received funding from the European Union’s Horizon 2020 research and innovation program under the Marie-Sklodowska-Curie grant agreement No 765329. G.H.S. received support from the NIHR Manchester Biomedical Research Centre.
